# Supplementary material for: Patterns of Intron Gain and Loss in Fungi
Source: PLoS Biol. 2004 Nov 30;2(12):e422. doi: 10.1371/journal.pbio.0020422 (PMC532390; doi:10.1371/journal.pbio.0020422)
Supplement: Table S1 — Also available at http://genes.mit.edu/NielsenEtAl/. (4.3 MB ZIP). [file pbio.0020422.st001.zip › NielsenEtAl/html/251.html]

AN2312.1.NCU06317.1.MG08808.1.FG05001.1


```
 CLUSTAL W (1.82) Multiple Sequence Alignments - Introns Inserted


Sequence 1: MG08808.1	157 aa
Sequence 2: FG05001.1	163 aa
Sequence 3: NCU06317.1	170 aa
Sequence 4: AN2312.1	157 aa
Alignment Length: 173 aa
Number Identitical Residues: 46 aa
Alignment Score (without introns) 2626


MG08808.1 	MCSADIFLGLLAILFPPLPV1WVKRGLCSADSLINILLCILGY0LPGLIHSWYIIARFPE
NCU06317.1	MWSTDIFLGFLAILFPPLPV1WVKTGICSADSFINLLLCVLGF~LPGLLHAWYIIAKNPT
FG05001.1 	MCSADFFLAFLAILFPPLPV1WVKCGLCSADSLINILLCCLGY~IPGLIHAWYLIAKYPE
AN2312.1  	MCGSDMFLAILAIFFPPVSV1WIKVGICTADSIINLALCCLGY~VPGLIHAWYIILKNPE
          	* .:*:**.:***:***:.* *:* *:*:***:**: ** **: :***:*:**:* : * 

MG08808.1 	EQYEYERG-YNRDAE-RVT-----YVFVAAN----GENQNSRGPARQPRPSANKSGAGPT
NCU06317.1	PPFDYDGAVYDANDIGRVESGQRIYVFVHDQP---GNNQTGGGRHYHHQQAAHKNQQPQR
FG05001.1 	PPYEYESLPQDREGN-RVT-----YVYVQCPPGPHQHGQNNKQPKPQPKPHSGNTGN-MN
AN2312.1  	PDYDDPSYQPVPNRRGDVENGHVTYYYVSHQP---APTRPQRGYGTMPSQHASDAQS---
          	  ::        :  . * ..   * :*   .      :            : .      

MG08808.1 	YGTTNNATNNQASNSRP---PQQTNAGEGGS--DGAAPPSYAQVVAGDNKIQTQD
NCU06317.1	QGPMNYGTTAGHAPQDSGIAHAGPSAGGDSDNSHAAPPPSYAQVVAGDHKIQTQD
FG05001.1 	YGTQNAASSSHPAPQQQ---QGVTNNGEGSSSNDQSVPPSYADVVAGDHKIQSKD
AN2312.1  	-------PSRPPAPKSQ---HEHNTAGSSSQANEDTSPPTYAEAVKGDHKVQNHD
          	       ..   : .         . * ... .. : **:**:.* **:*:*.:*
```
